# A Hydrothermal and Combustion-Reduction Process with Polyvinyl Pyrrolidone as a Restricted Growth Agent and Galactose as a Reducing Agent for the Fabrication of Rod-like α-Fe_2_O_3_/Fe_3_O_4_ Magnetic Nanocomposites

**DOI:** 10.3390/ma18051014

**Published:** 2025-02-25

**Authors:** Yuxuan Bai, Zhou Wang, Yongjin Li

**Affiliations:** 1 School of Medicine, Jiangsu University, Zhenjiang 212013, China; 3220902020@stmail.ujs.edu.cn; 2 College of Vanadium and Titanium, Panzhihua University, Panzhihua 617000, China

**Keywords:** α-Fe_2_O_3_/Fe_3_O_4_ MNCs, β-FeOOH nanorods, hydrothermal process, combustion-reduction process, PVP, galactose

## Abstract

A hydrothermal and combustion-reduction process with polyvinyl pyrrolidone (PVP) as a restricted growth agent and galactose as a reducing agent was developed for the fabrication of rod-like α-Fe_2_O_3_/Fe_3_O_4_ magnetic nanocomposites (MNCs). Firstly, β-FeOOH nanorods (NRs) were fabricated by the hydrothermal method, with PVP as a restricted growth agent. To obtain a smaller size for better applications in the biomedical field, the concentrations of FeCl_3_ and PVP, the hydrothermal temperature, and the hydrothermal time were optimized as 0.171 M, 0.163 mM, 100 °C, and 8 h, and the fabricated β-FeOOH NRs were 193.1 nm in average length and 43.2 nm in average diameter. Then, with β-FeOOH NRs as precursors, α-Fe_2_O_3_/Fe_3_O_4_ MNCs were prepared via the combustion-reduction process with galactose as a reducing agent; the factors of the calcination temperature and time and the mass ratio of β-FeOOH and galactose were assessed as 300 °C, 0.5 h, and 1:2, respectively. The prepared α-Fe_2_O_3_/Fe_3_O_4_ MNCs under the optimized conditions were 81.6 nm in average length and 23.9 nm in average diameter, while their saturation magnetization reached 69.8 emu/g.

## 1. Introduction

Many novel magnetic nanomaterials have experienced wider interest in the last decades owing to many excellent properties that differ from traditional materials, such as stable physical and chemical properties, satisfying surfaces, interface effects, a large specific surface area, and so on [1,2], and magnetic nanomaterials also obtain wide applications in various fields. For example, in the biomedical field, magnetic nanomaterials are particularly promising for their unique physicochemical properties that can support multiple functions, including X-ray computed tomography, cancer diagnosis by magnetic resonance imaging [3], Raman and photoacoustic imaging [4], drug delivery [5,6], the target therapy of cancers, and plasmonic photothermal and photodynamic therapies [7,8]. In the electrochemical detection area, magnetic nanomaterials can be applied as electrode modifiers and sorbents to enhance electrochemical signals for sensitive detection [9,10].

Among nanomaterials, hematite (α-Fe_2_O_3_) and magnetite (Fe_3_O_4_) are chemically stable, non-toxic, environmentally friendly, and have other unique characteristics, such as a high surface-to-volume ratio and so on [11]; they are often applied in biomedicine research. However, these magnetic materials have excessive or excessively low magnetic properties in practical applications [12]. To solve the above problem, α-Fe_2_O_3_/Fe_3_O_4_ magnetic nanocomposites (MNCs) have attracted our attention due to their appropriate magnetic properties for bio-applications [13,14]; among them, rod-like α-Fe_2_O_3_/Fe_3_O_4_ MNCs have especially become a research focus in biomedicine owing to their being devoured by cells and achieving easy entry into cells [2]; so, rod-like α-Fe_2_O_3_/Fe_3_O_4_ MNCs have caught researchers’ fancy.

α-Fe_2_O_3_/Fe_3_O_4_ MNCs can be fabricated by the following methods: the sonication/sol–gel method [15,16], the solvothermal method [17,18], incubation–calcination method [19], chemical vapor deposition and calcination process [20], solution combustion and calcination process [21], etc. These methods have drawbacks of complexity, a high cost, and harm to the environment. The proposed hydrothermal process with PVP as the restricted growth agent and combustion-reduction process with galactose as the reducing agent have the advantages of simple operation, practicability, high stability, and low cost [22,23].

In this work, a hydrothermal and combustion-reduction process was introduced for the preparation of α-Fe_2_O_3_/Fe_3_O_4_ MNCs with FeCl_3_ and polyvinyl pyrrolidone (PVP) as materials and galactose as a reducing agent, and the conditions of the process for the fabrication of α-Fe_2_O_3_/Fe_3_O_4_ MNCs were optimized in detail, and the fabrication mechanism was revealed.

## 2. Experimental

### 2.1. Materials

Polyvinyl Pyrrolidone (AR), anhydrous ferric chloride (AR), and galactose (AR) were purchased from Sinopharm Chemical Reagent Co., Ltd. (Shanghai, China), the absolute ethanol (AR) was purchased from Xilong Scientific Co., Ltd. (Shantou, China), LO2 cells and RPMI 1640 were purchased from Hefei Wanwu Biotechnology Co., Ltd. (Hefei, China).

### 2.2. Fabrication and Characterization of β-FeOOH Nanorods

A novel hydrothermal process with PVP as the restricted growth agent was developed to fabricate β-FeOOH nanorods (NRs). Typically, PVP and anhydrous ferric chloride were dissolved into 70 mL distilled water to form homogeneous solutions containing various concentrations of anhydrous ferric chloride (0.171 M, 0.342 M, 0.513 M, 0.684 M, and 0.855 M) and PVP (0 mM, 0.054 mM, 0.109 mM, 0.163 mM, 0.217 mM, and 0.271 mM), which were transferred to hydrothermal reactors, followed by being heated to different hydrothermal temperatures (40 °C, 60 °C, 80 °C, and 100 °C) for various hydrothermal times (2 h, 4 h, 6 h, 8 h, 10 h, and 12 h) in a furnace; after the hydrothermal reactions finished, the hydrothermal reactors were cooled to room temperature. The mixtures were centrifugally separated under 10,000 rpm/min in a centrifuge, and the obtained solids were alternately washed with ethanol and deionized water at least three times and dried for 12 h, and they were ground to obtain β-FeOOH NRs. The composition and microstructure of the β-FeOOH NRs were investigated by X-ray diffraction (XRD), under the conditions of a Cu-Kα ray with a scan rate of 5°/min for 2θ of 20–80°, and by scanning electron microscope (SEM).

### 2.3. Preparation and Characterization of α-Fe_2_O_3_/Fe_3_O_4_ MNCs

α-Fe_2_O_3_/Fe_3_O_4_ MNCs were prepared via the combustion-reduction process with galactose as a reducing agent. Firstly, β-FeOOH NRs and galactose were uniformly mixed according to their diverse mass ratios (1:1, 1:2, 1:4, 1:6, and 1:8), and placed into crucibles. Then, the mixtures together with the crucibles were calcined at various calcination temperatures (200–700 °C, with intervals of 100 °C) for different calcination times (0.5–2.0 h, with intervals of 0.5 h) in a programmed temperature furnace; the products were ground after being naturally cooled to room temperature, and rod-like α-Fe_2_O_3_/Fe_3_O_4_ MNCs were obtained. The composition, morphology, and microstructure of the α-Fe_2_O_3_/Fe_3_O_4_ MNCs were verified by XRD and transmission electron microscope (TEM), and their hysteresis loops were measured on a vibrating sample magnetometer (VSM).

### 2.4. MTT Assay

After the LO2 cells were resuscitated, the LO2 cells were cultured in RPMI 1640. When the fusion degree of the LO2 cells reached 80%, the LO2 cells were planted in 96-well plates. After 24 h, the α-Fe_2_O_3_/Fe_3_O_4_ MNCs were sterilized by ultraviolet lamp and dispersed in RPMI 1640 to form suspensions with various concentrations (0–1000 μg/mL), and then the suspensions were added into the plates, and 20 μL MTT solutions (5%) were added to the plates and cultured for another 24 h, and then the absorbances of the plates were measured. The viabilities of the LO2 cells were calculated.

## 3. Results and Discussion

### 3.1. Characteristics of β-FeOOH NRs

**Figure 1:**
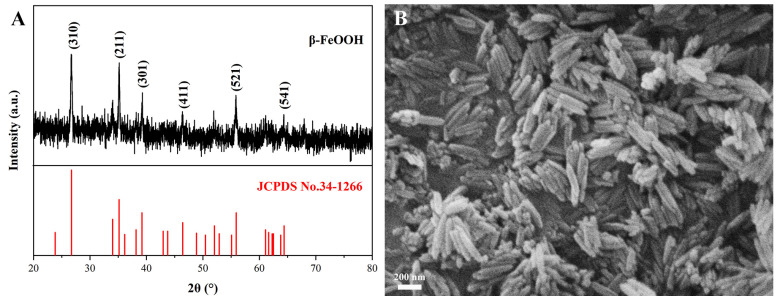
The XRD pattern (**A**) and SEM morphology (**B**) of β-FeOOH NRs fabricated at 100 °C for 8 h in 70 mL solution containing FeCl_3_ of 0.171 M and PVP of 0.163 mM.

The XRD pattern and SEM morphology of the β-FeOOH NRs fabricated at 100 °C for 8 h in a 70 mL solution containing FeCl_3_ of 0.171 M and PVP of 0.163 mM are revealed in Figure 1. It was found from the XRD pattern (Figure 1A) that the X-ray diffraction peaks at 26.7°, 35.1°, 39.2°, 46.4°, 55.9°, and 64.4° were perfectly fitted to the β-FeOOH standard PDF card (JCPDS No. 34-1266), which affirmed that the product belonged to the phase of β-FeOOH. Figure 1B shows their SEM morphology; obviously, the structures of the products were nanorods, and their average diameter and length reached 43.2 nm and 193.1 nm, respectively. All the above results demonstrated that β-FeOOH NRs were successfully fabricated.

### 3.2. Optimization of Fabrication Conditions for β-FeOOH NRs

During the hydrolysis process, FeCl_3_ was thermally decomposed to HCl and Fe(OH)_3_, and then the as-generated Fe(OH)_3_ continued to decompose and formed β-FeOOH. PVP acted as a dispersant and adsorbed on the β-FeOOH crystal nucleus through the coordination bond formed between the lone pair electrons of nitrogen or oxygen atoms in its structure and the atoms on the β-FeOOH surface, which enabled the C-H chain to fully expand in water and spread out in all directions. Therefore, the C-H chain prevented aggregation between the nanorod and made the β-FeOOH crystal nucleus grow in one direction and form β-FeOOH NRs, and it made them uniformly dispersed in the solution. Therefore, the effects of the FeCl_3_ concentration, PVP concentration, hydrothermal temperature, and hydrothermal time were the key factors, so all the preparation conditions were optimized.

#### 3.2.1. Optimization of FeCl_3_ Concentration

**Figure 2:**
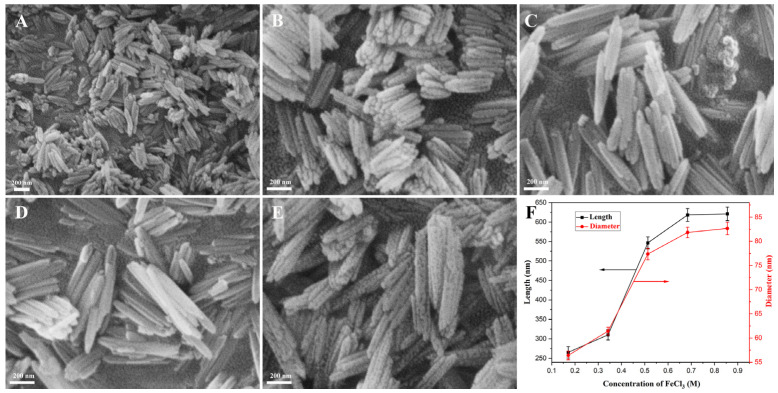
The SEM morphologies of β-FeOOH NRs fabricated at 100 °C for 10 h in each 70 mL solution containing FeCl_3_ of 0.171 M (**A**), 0.342 M (**B**), 0.513 M (**C**), 0.684 M (**D**), and 0.855 M (**E**) and PVP of 0.271 mM, and the corresponding broken-line graph of dimension change (**F**).

The solutions containing 0.054 mM PVP and FeCl_3_ of various concentrations were hydrothermally treated at 100 °C for 10 h; when the concentration of FeCl_3_ was below 0.088 M, the product was almost unobtained; the other yields are listed in Table 1, and the microstructures of the as-prepared β-FeOOH NRs fabricated with various concentrations of FeCl_3_ (0.171–0.855 M) and their size change rules are displayed in Figure 2. Obviously, the products prepared with various FeCl_3_ concentrations were rod-like in structure; their average lengths and diameters are shown in Figure 2F; with FeCl_3_ concentration increasing from 0.171 M to 0.855 M, their average length increased from 265.5 nm to 621.1 nm, while their average diameter increased from 56.4 nm to 82.7 nm. The reason for this was that the low Fe^3+^ concentration mainly affected the growth degree of the crystals; the increase in Fe^3+^ concentration provided more raw materials for the growth of β-FeOOH crystals, and it made their particle size larger and the crystallinity stronger. For a smaller size and better applications in biomedicine, the optimal concentration of FeCl_3_ was selected as 0.171 M.

#### 3.2.2. Optimization of PVP Concentration

**Figure 3:**
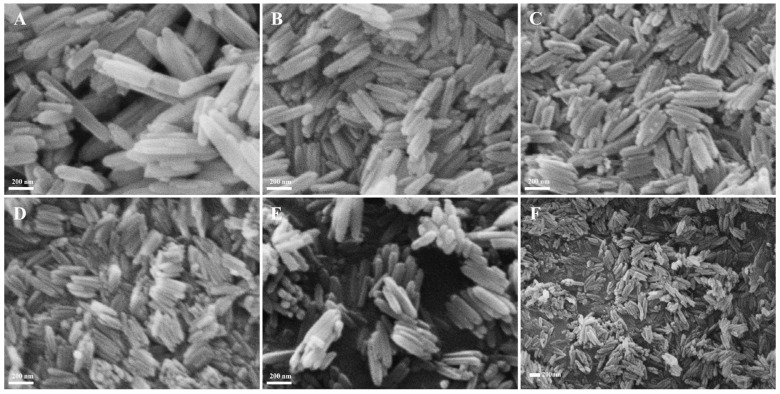
The SEM morphologies of β-FeOOH NRs fabricated at 100 °C for 10 h in each 70 mL solution containing FeCl_3_ of 0.171 M and PVP of 0 mM (**A**), 0.054 mM (**B**), 0.109 mM (**C**), 0.163 mM (**D**), 0.217 mM (**E**), and 0.271 mM (**F**).

**Figure 4:**
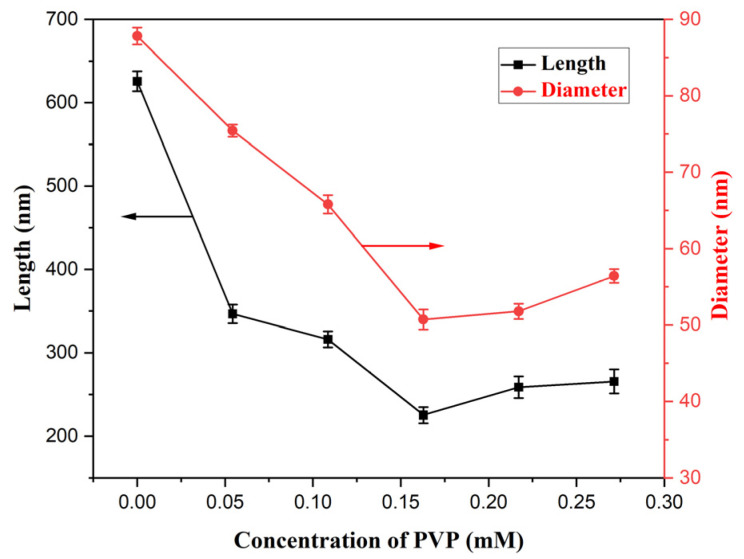
Broken-line graph of dimension change with various PVP concentrations.

The solutions containing 0.171 M FeCl_3_ and various PVP concentrations experienced hydrothermal reaction at 100 °C for 10 h; the SEM morphologies of the products are revealed in Figure 3. While all the products displayed a nanorod structure, their average diameter and length were largest at 87.8 nm and 625.7 nm without PVP in the solution (Figure 3A); with the PVP concentration increasing from 0.054 mM to 0.163 mM (Figure 3B–D), their average length decreased from 346.5 nm to 225.2 nm, while their average diameter also decreased from 75.4 nm to 50.7 nm, and their change trend is revealed in Figure 4, which obviously demonstrates the role of restrictive growth and the dispersion effect of PVP. With the continued increase in PVP concentration, their average diameter and length almost stayed immovable. The corresponding results likewise are listed in Table 1. Therefore, for the same purpose, the concentration of PVP was selected as 0.163 mM for the fabrication of β-FeOOH NRs.

#### 3.2.3. Optimization of Hydrothermal Temperature

**Figure 5:**
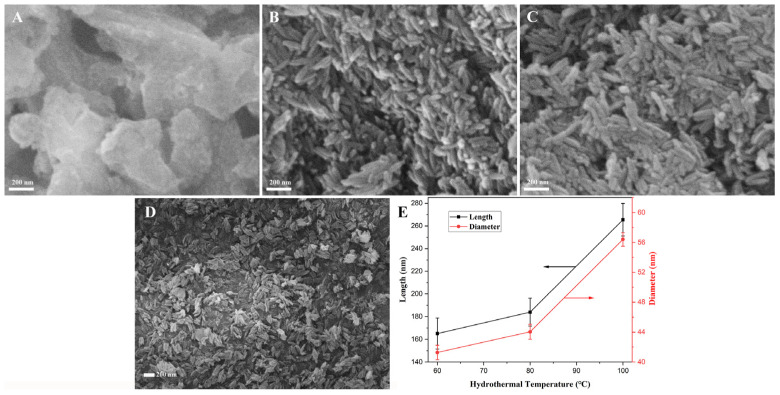
The SEM morphologies of β-FeOOH NRs fabricated at 40 °C (**A**), 60 °C (**B**), 80 °C (**C**), and 100 °C (**D**) for 10 h in each 70 mL solution containing FeCl_3_ of 0.171 M and PVP of 0.271 mM, and the corresponding broken-line graph of dimension change (**E**).

The solutions of 70 mL containing 0.171 M FeCl_3_ and 0.163 mM PVP reacted at various temperatures for 10 h via the hydrothermal process; the SEM morphologies of the as-prepared products are revealed in Figure 5. From Figure 5A, when the hydrothermal treatment was 40 °C, the β-FeOOH NRs were unformed, while rod-shaped β-FeOOH precursors with a well-arranged and good morphology were formed, as in Figure 5B–D, as the hydrothermal temperature was enhanced from 60 °C to 100 °C. Their average length increased from 165.1 nm and 265.5 nm, while the average diameter increased from 41.3 nm and 56.4 nm, which is shown in Figure 5E; with the increase in hydrothermal temperature, the morphology of the nanorods became uniform, and the size increased gradually. The reason for this was that the solubility of the FeCl_3_ increased with the rise in hydrothermal temperature; then, the concentration of Fe^3+^ in the solution expanded, which was advantageous to the growth of the crystals. The yield (Table 1) of β-FeOOH NRs increased rapidly from 0.91% to 28.19% with the rise in hydrothermal temperature. According to the above the results, the optimum hydrothermal temperature was selected as 100 °C for the fabrication of β-FeOOH NRs.

#### 3.2.4. Optimization of Hydrothermal Time

**Figure 6:**
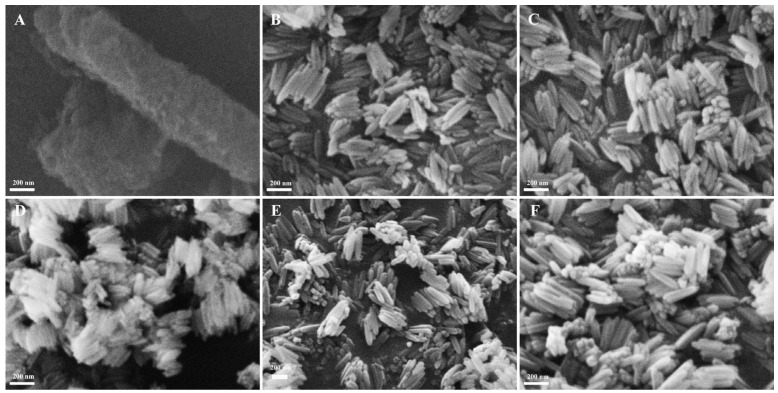
The SEM morphologies of β-FeOOH NRs experiencing a hydrothermal reaction at 100 °C for 2 h (**A**), 4 h (**B**), 6 h (**C**), 8 h (**D**), 10 h (**E**), and 12 h (**F**) with FeCl_3_ of 0.171 M and PVP of 0.217 mM.

**Figure 7:**
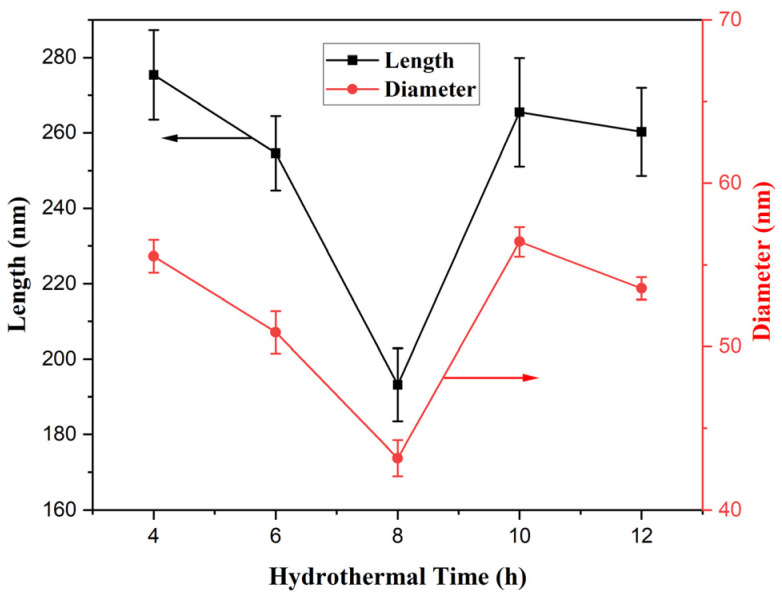
Broken-line graph of dimension change against hydrothermal time.

The SEM morphologies of the products fabricated at 100 °C for different hydrothermal times in each 70 mL solution containing 0.171 M FeCl_3_ and 0.163 mM PVP are shown in Figure 6; obviously, the products fabricated for hydrothermal times of 4–12 h were rod-structured, but they failed to form rod structures for hydrothermal time of 2 h. At the same time, the average diameter and length of the β-FeOOH NRs decreased with the time prolonging from 2 h to 8 h and then increased when the time continued to prolong; the experimental data are shown in Figure 7. The reason might be that the extension of time was advantageous to the dehydration reaction of Fe(OH)_3_, and β-FeOOH NRs with a short length and uniform morphology were produced. The yield of β-FeOOH NRs increased first and then decreased with the increase in hydrothermal time, as listed in Table 1. According to the yield and the size of the nanorods, the optimal hydrothermal time was 8 h.

In conclusion, for a larger yield, faster production speed, and smaller size, the optimal preparation conditions for β-FeOOH NRs were an FeCl_3_ concentration of 0.171 M, a PVP concentration of 0.163 mM, a hydrothermal time of 8 h, and a hydrothermal temperature of 100 °C.

### 3.3. Characteristics and MTT Evaluation of α-Fe_2_O_3_/Fe_3_O_4_ MNCs

**Figure 8:**
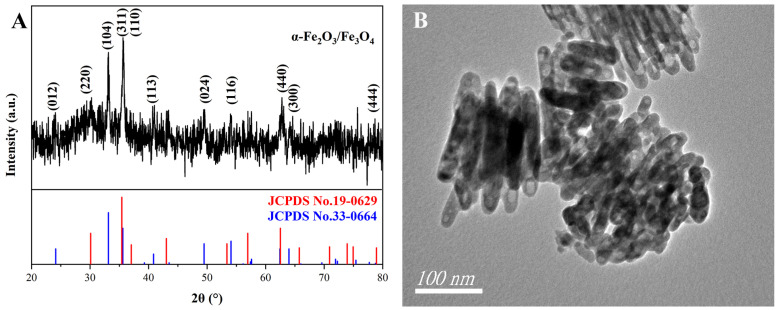
XRD pattern (**A**) and TEM image (**B**) of α-Fe_2_O_3_/Fe_3_O_4_ MNCs.

Taking β-FeOOH NRs as precursors, α-Fe_2_O_3_/Fe_3_O_4_ MNCs were prepared via the combustion-reduction process with galactose as a reducing agent. Figure 8 shows the XRD pattern and the TEM image of α-Fe_2_O_3_/Fe_3_O_4_ MNCs calcined at 300 °C for 1.5 h with a mass ratio of 1:2 for β-FeOOH NRs and galactose. The XRD pattern (Figure 8A) showed that the X-ray diffraction peaks at a 2θ of 30.1°, 35.4°, 62.5°, and 78.9° corresponded to the Fe_3_O_4_ standard card (JCPDS No. 19-0629), while the X-ray diffraction peaks at 24.1°, 33.2°, 35.6°, 40.8°, 49.5°, 54.1°, and 64.0° corresponded to the standard card of α-Fe_2_O_3_ (JCPDS No. 33-0664), which confirmed the existence of α-Fe_2_O_3_ and Fe_3_O_4_ phases in the product. The TEM image (Figure 8B) confirmed the rod-like structure of the α-Fe_2_O_3_/Fe_3_O_4_ MNCs, with an average diameter and length of 23.9 nm and 81.6 nm.

**Figure 9:**
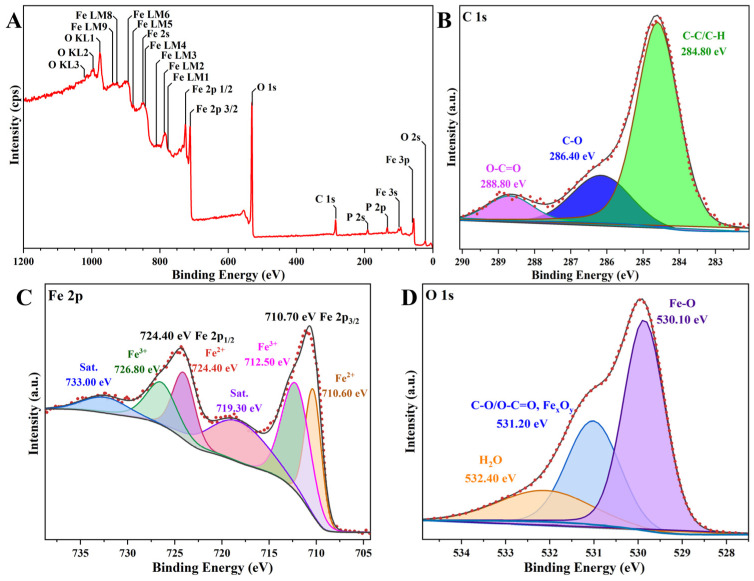
The XPS spectrum (**A**) and high-resolution XPS spectra (**B**–**D**) of F α-Fe_2_O_3_/Fe_3_O_4_ MNCs calcined at 300 °C for 1.5 h with a mass ratio of 1:2 for β-FeOOH NRs and galactose.

The surface element composition, chemical state, and electronic state of the α-Fe_2_O_3_/Fe_3_O_4_ MNCs were analyzed by the XPS method, and their XPS spectra are displayed in Figure 9. Firstly, all the peaks were calibrated using the binding energy (BE) of C 1s in the vacuum system (284.80 eV) as a reference [24]. As shown in Figure 9A, the BE of the C 1s peak is 284.85 eV; thus, the C 1s, Fe 2p, and O 1s peaks were calibrated with 0.05 eV of the chemical shift, as shown in Figure 9B–D. Figure 9C presents the high-resolution XPS spectrum of Fe 2p; the characteristic peaks appeared at BEs of approximately 710.70 eV and 724.40 eV, corresponding to Fe 2p_3/2_ and Fe 2p_1/2_, respectively [25], and the peaks around 719.10 eV and 732.80 eV belonged to the satellite peaks, which arose from the charge transfer screening. Upon deconvolving the Fe 2p characteristic peaks via Gaussian–Lorentz profile patterns, it was determined that the splitting peaks at high BEs of around 712.30 eV and 726.60 eV were attributed to Fe(III), while the splitting peaks at approximately 710.4 eV and 724.2 eV were ascribable to Fe(II). All the results demonstrated the formation of α-Fe_2_O_3_/Fe_3_O_4_ MNCs due to the existences of Fe(II) and Fe(III) [26,27]. The narrow spectrum of O 1s is presented in Figure 9D [28]. All the above results demonstrated again the successful formation of α-Fe_2_O_3_/Fe_3_O_4_ MNCs.

**Figure 10:**
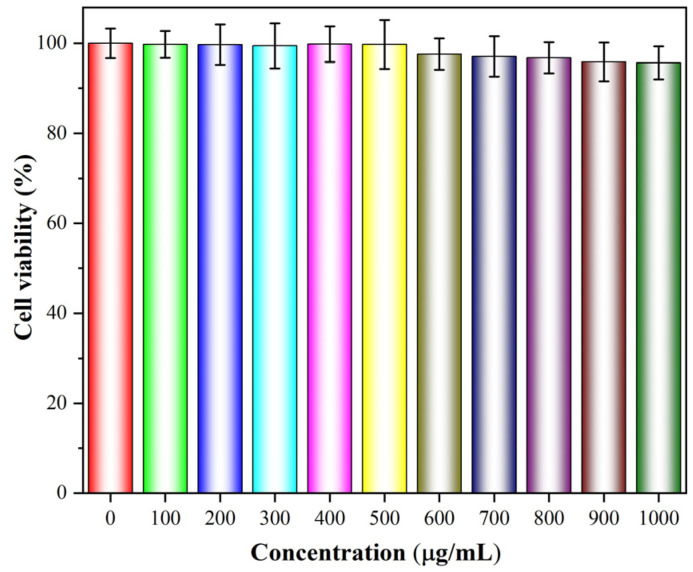
The cell viabilities of LO2 cells incubated with various concentrations of α-Fe_2_O_3_/Fe_3_O_4_ MNCs for 24 h (*n* = 3).

Figure 10 displays the cell viabilities of LO2 affected by various concentrations of α-Fe_2_O_3_/Fe_3_O_4_ MNCs (0–1000 μg/mL) for 24 h. Compared with the blank group (0 μg/mL of α-Fe_2_O_3_/Fe_3_O_4_ MNCs suspension), all the cell viabilities of LO2 cells affected by α-Fe_2_O_3_/Fe_3_O_4_ MNCs suspension of 100–1000 μg/mL were not lower than 95.6% of those of LO2 cells affected by a suspension without α-Fe_2_O_3_/Fe_3_O_4_ MNCs, which revealed α-Fe_2_O_3_/Fe_3_O_4_ MNCs showed excellent biocompatibility and lower toxicity.

### 3.4. Optimization of Preparation Conditions for α-Fe_2_O_3_/Fe_3_O_4_ MNCs

#### 3.4.1. Optimization of Calcination Temperature

**Figure 11:**
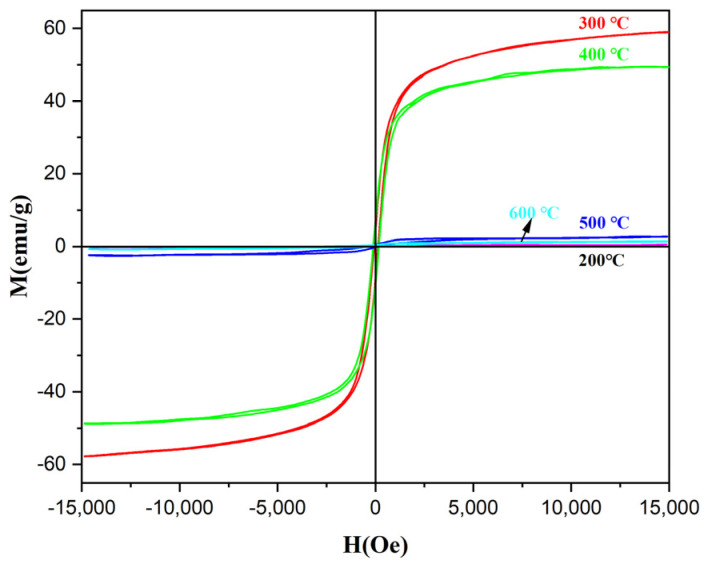
The hysteresis loops of α−Fe_2_O_3_/Fe_3_O_4_ MNCs calcined at diverse temperatures for 2 h with a mass ratio of 1:6 for β−FeOOH NRs and galactose.

Figure 11 displays the hysteresis loops of α-Fe_2_O_3_/Fe_3_O_4_ MNCs calcined at different temperatures for 2 h with a 1:6 mass ratio for β-FeOOH NRs and galactose; the α-Fe_2_O_3_/Fe_3_O_4_ MNCs began to reveal the characteristic of superparamagnetism when the calcination temperature achieved 200 °C, which suggested that α-Fe_2_O_3_/Fe_3_O_4_ MNCs began to form. However, for the lowest the saturation magnetization (Ms) for α-Fe_2_O_3_/Fe_3_O_4_ MNCs calcined at 200 °C, the reason might be that the calcination temperature was below the thermal decomposition temperature of galactose, which affected the growth of the crystals. Therefore, the reduction of galactose did not take place completely. As the calcination temperature increased to 300 °C, the reduction of galactose was more obvious, the content of the Fe_3_O_4_ phase in α-Fe_2_O_3_/Fe_3_O_4_ MNCs increased gradually, and the color of the product changed from red to black; the Ms of α-Fe_2_O_3_/Fe_3_O_4_ MNCs achieved the maximum value of 59.0 emu/g. However, as the calcination temperature exceeded 300 °C, galactose began to decompose at express speed; when the galactose was exhausted, the reduction effect decreased, the reduced Fe_3_O_4_ began to be oxidized to α-Fe_2_O_3_, and the content of the α-Fe_2_O_3_ phase in α-Fe_2_O_3_/Fe_3_O_4_ MNCs increased gradually, so their Ms value decreased. When the temperature exceeded 500 °C, Fe_3_O_4_ was almost oxidized to α-Fe_2_O_3_ completely due to the enhancement of oxidation at high temperature, and then single-phase α-Fe_2_O_3_ nanorods with a low saturation magnetization were obtained. For larger Ms and better applications in biomedicine, the optimal calcination temperature was selected as 300 °C.

#### 3.4.2. Optimization of Calcination Time

**Figure 12:**
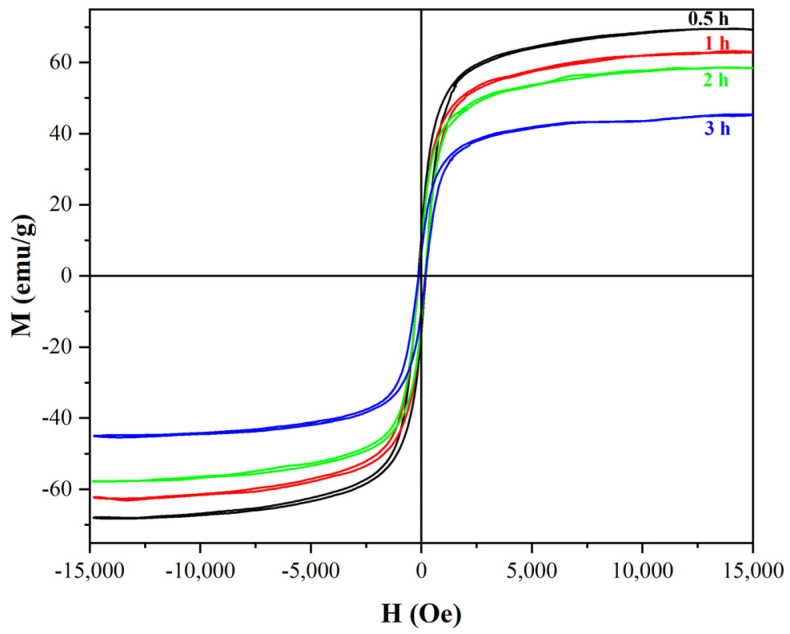
The hysteresis loops of α−Fe_2_O_3_/Fe_3_O_4_ MNCs calcined at 300 °C for diverse times with a mass ratio of 1:6 for β−FeOOH NRs and galactose.

Figure 12 displays the hysteresis loops of α-Fe_2_O_3_/Fe_3_O_4_ MNCs calcined at 300 °C for various times, with a mass ratio of 1:6 for β-FeOOH NRs and galactose. Obviously, with the calcination time prolonging from 0.5 h to 2.0 h, the Ms of α-Fe_2_O_3_/Fe_3_O_4_ MNCs continually decreased, which revealed that the extension of the calcination time resulted in the deep oxidation of the nanorods and the phase transition from Fe_3_O_4_ to α-Fe_2_O_3_. Therefore, 0.5 h was employed as the optimal calcination time, and the maximal Ms of α-Fe_2_O_3_/Fe_3_O_4_ MNCs could reach 69.4 emu/g.

#### 3.4.3. Optimization of Mass Ratio for β-FeOOH NRs and Galactose

**Figure 13:**
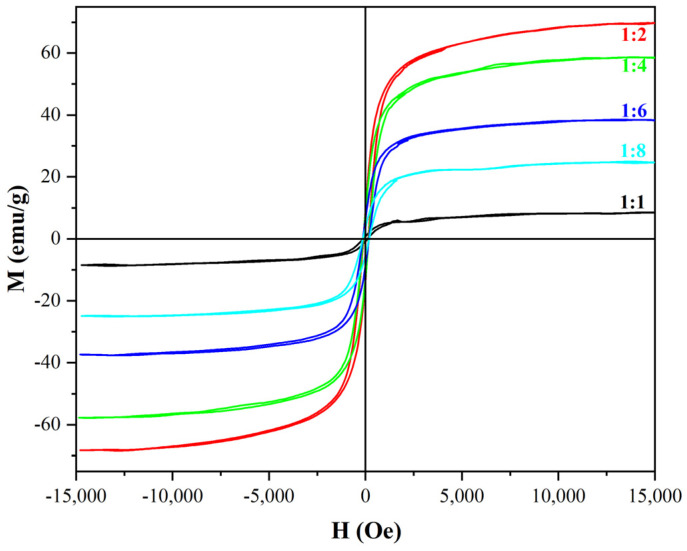
The hysteresis loops of α−Fe_2_O_3_/Fe_3_O_4_ MNCs calcined at 300 °C for 0.5 h with diverse mass ratios of β−FeOOH NRs and galactose.

Figure 13 reveals the hysteresis loops of α-Fe_2_O_3_/Fe_3_O_4_ MNCs calcined at 300 °C for 0.5 h with diverse mass ratios for β-FeOOH NRs and galactose. The Ms were enhanced with the mass ratio decreasing from 1:1 to 1:2. However, when the mass ratio continued to decrease to 1:8, galactose was excessive due to the limitations of the calcination temperature and time, the content of Fe_3_O_4_ in α-Fe_2_O_3_/Fe_3_O_4_ MNCs reduced, so the saturation magnetization also decreased. Therefore, for playing the role of a magnetic property in applications, the mass ratio 1:2 was selected as the optimal ratio for the preparation of α-Fe_2_O_3_/Fe_3_O_4_ MNCs, while the Ms of the prepared α-Fe_2_O_3_/Fe_3_O_4_ MNCs under optimal conditions reached 69.8 emu/g.

To sum up, for smaller size and larger Ms, the mixture of β-FeOOH NRs and galactose could be calcined at 300 °C for 0.5 h with a mass ratio of 1:2 to prepare rod-like α-Fe_2_O_3_/Fe_3_O_4_ MNCs.

## 4. Conclusions

A hydrothermal process was developed to fabricate β-FeOOH NRs, and the optimal fabrication conditions were an FeCl_3_ concentration of 0.171 M, a PVP concentration of 0.163 mM, a hydrothermal time of 8 h, and a hydrothermal temperature of 100 °C; the average diameter and length of the fabricated β-FeOOH NRs were 43.2 nm and 193.1 nm, respectively.

α-Fe_2_O_3_/Fe_3_O_4_ MNCs were prepared via the combustion-reduction process with galactose as a reducing agent. For a smaller size and larger Ms, the mixture of β-FeOOH NRs and galactose could be calcined at 300 °C for 0.5 h with a mass ratio of 1:2 to prepare rod-like α-Fe_2_O_3_/Fe_3_O_4_ MNCs, and the average diameter and length of the as-prepared rod-like α-Fe_2_O_3_/Fe_3_O_4_ MNCs were 23.9 nm and 81.6 nm, and their Ms could reach 69.8 emu/g.

## Figures and Tables

**Table 1:** Productivities of β-FeOOH precursors fabricated under various conditions.

FeCl_3_ (M)	PVP (mM)	Hydrothermal Time (h)	Hydrothermal Temperature (°C)	Yield (%)
0.171	0.271	10	100	28.19
0.342	0.271	10	100	14.63
0.513	0.271	10	100	8.90
0.684	0.271	10	100	7.27
0.855	0.271	10	100	5.25
0.171	0.271	10	80	24.30
0.171	0.271	10	60	6.91
0.171	0.271	10	40	0.91
0.171	0.000	10	100	25.19
0.171	0.054	10	100	28.10
0.171	0.109	10	100	28.56
0.171	0.163	10	100	30.13
0.171	0.217	10	100	29.31
0.171	0.163	2	100	1.47
0.171	0.163	4	100	23.87
0.171	0.163	6	100	26.66
0.171	0.163	8	100	33.31
0.171	0.163	12	100	33.19

## Data Availability

The original contributions presented in this study are included in the article. Further inquiries can be directed to the corresponding author.

## References

[1] Wang X.Y., Zhang X.J., Zhao S.H., Zhou J.Y., Wu L.P., Liu R.J. (2024). Fabrication and Characterization of Magnetic Fe_3_O_4_/α-Fe_2_O_3_ Heterogeneous Nanorods. J. Inorg. Organomet. Polym..

[2] Zhu Z.Y., Ouyang H.Z., Ling C., Ma M.Y., Wang J., Yu X., Li Y.J. (2023). Fabrication of Magnetic α-Fe_2_O_3_/Fe_3_O_4_ Heterostructure Nanorods via the Urea Hydrolysis-Calcination Process and Their Biocompatibility with LO_2_ and HepG_2_ Cells. Nanotechnology.

[3] Ju Q., Huang R., Hu R.M., Fan J.J., Zhang D.L., Ding J., Li R. (2023). Phytic Acid-Modified Manganese Dioxide Nanoparticles Oligomer for Magnetic Resonance Imaging and Targeting Therapy of Osteosarcoma. Drug Deliv..

[4] Liu L., Yang S.P., Zheng Z.L., Li Q.S., Liu C.C., Hu D.H., Liu Z., Zhang X.P., Zhang R.P., Gao D.Y. (2023). Biomimetic Theranostic Agents with Superior NIR-II Photoacoustic and Magnetic Resonance Imaging Performance for Targeted Photothermal Therapy of Prostate Cancer. Pharmaceutics.

[5] Ni Y., Deng P., Yin R.T., Zhu Z.Y., Ling C., Ma M.Y., Wang J., Li S.S., Liu R.J. (2023). Effect and Mechanism of Paclitaxel Loaded on Magnetic Fe_3_O_4_@mSiO_2_-NH_2_-FA Nanocomposites to MCF-7 Cells. Drug Deliv..

[6] Trucillo P. (2024). Biomaterials for Drug Delivery and Human Applications. Materials.

[7] Koshev N., Kapralov P., Evstigneeva S., Lutsenko O., Shilina P., Zharkov M., Pyataev N., Darwish A., Timin A., Ostras M. (2024). Yttrium-Iron Garnet Film Magnetometer for Registration of Magnetic Nano- and Submicron Particles: In Vitro and In Vivo Studies. IEEE T. Biomed. Eng..

[8] Ahmadi M., Ghoorchian A., Dashtian K., Kamalabadi M., Madrakian T., Afkhami A. (2021). Application of Magnetic Nanomaterials in Electroanalytical Methods: A Review. Talanta.

[9] Yue Y., Zhang X.J., Zhao S.H., Wang X.Y., Wang J., Liu R.J. (2024). Construction of a Label-Free Electrochemical Biosensing System Utilizing Fe_3_O_4_/α-Fe_2_O_3_@Au with Magnetic-Induced Self-Assembly for the Detection of EGFR Glycoprotein. Vacuum.

[10] Zhang Y.L., Wang J., Liu M., Ni Y., Yue Y., He D.W., Liu R.J. (2024). Magnetically Induced Self-Assembly Electrochemical Biosensor with Ultra-Low Detection Limit and Extended Measuring Range for Sensitive Detection of HER2 Protein. Bioelectrochemistry.

[11] Rytov R.A., Usov N.A. (2023). Specific Absorption Rate of Randomly Oriented Magnetic Nanoparticles in a Static Magnetic Field. Beilstein J. Nanotechnol..

[12] Wang J., Liu M., Tang J.W., Yang Y.P., He N., Li S.S., Liu R.J. (2024). Construction of Electrochemical Biosensor Based on Magnetic Fe_3_O_4_/α-Fe_2_O_3_ Heterogeneous Nanorods for the Sensitive Detection of MUC1 Mucoprotein. Ceram. Int..

[13] Wang J., Ouyang H.Z., Ni Y., Zhang H.D., Sun L., Liu R.J., Li S.S. (2024). Magnetic Self-Assembled Label-Free Electrochemical Biosensor Based on Fe_3_O_4_/α-Fe_2_O_3_ Heterogeneous Nanosheets for the Detection of Tau Proteins. Bioelectrochemistry.

[14] Yue Y., Zhang X.J., Xu Z.H., Sun L., Li S.S., Liu R.J. (2024). Ultrasensitive Detection of PSA in Human Serum Using Label-Free Electrochemical Biosensor with Magnetically Induced Self-Assembly Based on α-Fe_2_O_3_/Fe_3_O_4_@Au Nanocomposites. Microchem. J..

[15] Nagare A., Dhadage A., Baithy M., Bhuyan P.M., Gogoi P., Athare A., Navgire M. (2024). Sol-Gel Assisted β-Cyclodextrin Coated MoO_3_-Fe_2_O_3_ Nanocomposite for Photodegradation of Methylene Blue Dye. J. Sol-Gel Sci. Technol..

[16] Kumar N., Banerjee A.M., Pai M.R., Meena S.S., Patra A.K., Sastry P.U., Jagannath, Tripathi A.K. (2023). Sol-Gel Mediated Synthesis and Characterization of Hierarchically Porous Fe_2_O_3_/SiO_2_ Monolithic Catalyst for High Temperature Sulfuric Acid Decomposition. Catal. Commun..

[17] Kanimozhi G., Nibagani N., Nair D.S., Kumar H., Satyanarayana N. (2022). A Synergic Effect of N-Graphene Wrapped α-Fe_2_O_3_ Nanofacets Prepared by Microwave-Assisted Solvothermal Method for Lithium-Ion Battery. J. Phys. Chem. Solids.

[18] Li P.H., Zhuang X.Y., Xu J.H., Ruan L.X., Jiang Y.F., Lin J.X., Zhang X.M. (2022). Enhanced Photo-Fenton Activity of SnO_2_/α-Fe_2_O_3_ Composites Prepared by a Two-Step Solvothermal Method. Materials.

[19] Srivastava N., Srivastava M., Alhazmi A., Mohammad A., Khan S., Pal D.B., Haque S., Singh R., Mishra P.K., Gupta V.K. (2021). Sustainable green approach to synthesize Fe_3_O_4_/α-Fe_2_O_3_ nanocomposite using waste pulp of *Syzygium cumini* and its application in functional stability of microbial cellulases. Sci. Rep..

[20] Leduc J., Goenuellue Y., Ghamgosar P., You S., Mouzon J., Choi H., Vomiero A., Grosch M., Mathur S. (2019). Electronically-Coupled Phase Boundaries in α-Fe_2_O_3_/Fe_3_O_4_ Nanocomposite Photoanodes for Enhanced Water Oxidation. ACS Appl. Nano Mater..

[21] Alkanad K., Hezam A., Shekar G.C.S., Drmosh Q.A., Kala A.L.A., AL-Gunaid M.Q.A., Lokanath N.K. (2021). Magnetic recyclable α-Fe_2_O_3_–Fe_3_O_4_/Co_3_O_4_–CoO nanocomposite with a dual Z-scheme charge transfer pathway for quick photo-Fenton degradation of organic pollutants. Catal. Sci. Technol..

[22] Liu R.J., Huang W., Pan S., Li Y., Yu L.L., He D.W. (2020). Covalent Immobilization and Characterization of Penicillin G Acylase on Magnetic Fe_2_O_3_/Fe_3_O_4_ Heterostructure Nanoparticles Prepared via a Novel Solution Combustion and Gel Calcination Process. Int. J. Biol. Macromol..

[23] Hong J.Z., Yang F., Sun Z.P. (2021). Hexagonal Bi-Pyramid α-Fe_2_O_3_ Microcrystals: Unusual Formation, Characterization and Application for Gas Sensing. J. Alloy. Compd..

[24] Xu Z.H., Lv Z.X., Yang H.J., Zhang J.S., Sun Z.J., He D.W., Liu R.J. (2025). Label-Free Electrochemical Biosensor with Magnetic Self-Assembly Constructed via PNA-DNA Hybridization Process on α-Fe_2_O_3_/Fe_3_O_4_ Nanosheets for APOE Ε4 Genes Ultrasensitive Detection. Bioelectrochemistry.

[25] Zhang S.H., Fan X.L., Xue J. (2023). A Novel Magnetic Manganese Oxide Halloysite Composite by One-Pot Synthesis for the Removal of Methylene Blue from Aqueous Solution. J. Alloy. Compd..

[26] Yamashita T., Hayes P. (2008). Analysis of XPS Spectra of Fe^2+^ and Fe^3+^ Ions in Oxide Materials. Appl. Surf. Sci..

[27] Zhu Z.Q., Huang Q.S. (2022). In-Plane Structured Fe_3_O_4_/FeS Composite Loaded on Reduced Graphene Oxide as a Stabilized Anode Material for Lithium-Ion Batteries. Appl. Phys. A.

[28] Li L., Ma P., Hussain S., Jia L.J., Lin D., Yin X., Lin Y., Cheng Z.H., Wang L.Y. (2019). FeS_2_/Carbon Hybrids on Carbon Cloth: A Highly Efficient and Stable Counter Electrode for Dye-Sensitized Solar Cells. Sustain. Energy Fuels.

